# The subject-environment interplay between runners from different Brazilian macro-regions

**DOI:** 10.3389/fpsyg.2023.1134797

**Published:** 2023-09-22

**Authors:** Mabliny Thuany, Paulo Felipe Ribeiro Bandeira, Douglas Vieira, Katja Weiss, Beat Knechtle, Thayse Natacha Gomes

**Affiliations:** ^1^Centre of Research, Education, Innovation and Intervention in Sport (CIFI2D), Faculty of Sport, University of Porto, Porto, Portugal; ^2^Department of Physical Education, Regional University of Cariri, Crato, Ceará, Brazil; ^3^Federal University of Vale do São Francisco—UNIVASF, Pernambuco, Brazil; ^4^Post-Graduation Program of Physical Education, Department of Physical Education, Federal University of Sergipe, São Cristóvão, Sergipe, Brazil; ^5^Institute of Primary Care, University of Zurich, Zürich, Switzerland; ^6^Medbase St. Gallen Am Vadianplatz, St. Gallen, Switzerland; ^7^Department of Physical Education and Sport Sciences, University of Limerick, Limerick, Ireland

**Keywords:** complex systems, endurance, exercise, network analysis, amateur athletes

## Abstract

**Purpose:**

Our purpose was to investigate the interplay between runners and their environment using a network approach.

**Methods:**

This cross-sectional study sampled Brazilian runners of both sexes, from the five macro-regions of the country. An electronic questionnaire was used to obtain information regarding age, sex, training volume, socio-economic level, place of residence, and running pace. Environmental indicators (public illumination, pavement, sidewalk, and green areas) were collected from available public information. Descriptive statistics were presented in mean (SD), and frequency (%). A network analysis was performed to evaluate the association between individual and environmental characteristics. Statistical analyses were performed in the JASP, considering *p* < 0.05.

**Results:**

At North and Mid-West regions, public illumination presents the highest values for the expected influence (1.74 and 1.56), while in Northeast and Southeast, sidewalks present the highest values (2.13; 0.91). For betweenness centrality, in North, Northeast, and Mid-West regions, residency in the capital of a state presented a hub. In contrast, pavement, and training volume present higher values in the South and Southeast. Network topologies are different.

**Conclusion:**

Public illumination (North and Mid-West) and sidewalk (Northeast, Southeast) were the most important variables for runners. Continental size countries need specific approaches to improve physical activity levels and health outcomes that consider the cultural, historical, and environmental background.

## 1. Introduction

Complex systems comprise the dynamic and non-linear relationships established between variables (Hristovski et al., 2012). From this relationship, a non-deterministic and irreducible behavior emerges (IPEA, 2015). Complex systems can be studied in different fields, including–but not limited to–biology (Cohen et al., 2022), physics (Robinson et al., 2022), psychology (Medin, 2017; Hayes and Andrews, 2020), and sports science (Pereira et al., 2018; Renfree and Casado, 2018). In the sports context, the complex systems approach was previously used to understand the subject-environment-task relationship in soccer players (Pol et al., 2020), performance predictors (Pereira et al., 2018; Renfree and Casado, 2018), and physical activity behaviors (PA) (Orr et al., 2016).

The association between the subject and environment has been highlighted as an important attribute of increasing physical activity levels (Smith et al., 2017; Tcymbal et al., 2020). In addition, the outdoor environment, comprising both the built and natural environments, has also been related to physical activities and health outcomes (Coon et al., 2011; Deelen et al., 2019). Individuals regularly engaged in outdoor physical activity presented lower values for somatic anxiety than those participating in indoor activities (Lawton et al., 2017). Moreover, green spaces were positively related to sports practice in high-income countries (i.e., China) (Wang et al., 2019). Despite the results of the studies being summarized at countries level, the generalization of results found in specific contexts is limited. These challenges are related to the cross-national differences experienced within countries.

For example, based on its territorial dimension, Brazil is a large country, with a political-geographical division into five macro-regions (i.e., North, Northeast, South, Southeast, and Mid-West) (Pokshishevskiy, 1960). The five macro-regions present specific characteristics regarding weather, economic development (Pokshishevskiy, 1960), cultural and lifestyle habits (Torres and Dessen, 2008), physical activity preferences (Brasil, 2015), and physical environment for outdoor activities (Hino et al., 2018). At a national level, the physical environment related to physical activity differs. Hino et al. (2018), reported that access spaces to leisure and sports practice, road structures for physical activity (sidewalks, illumination, and pavement), and tree-lined streets can influence the population involvement in physical activity. The authors also showed that South (i.e., Curitiba) and Southeast regions (i.e., São Paulo, Minas Gerais, Espírito Santo) presented better indicators related to the built environment, as well as a friendlier environment for physical activity compared to other regions (Hino et al., 2018), due to the higher indicators for green spaces, illumination, training facilities, and paved streets (Galatti, 2017; Hino et al., 2018). These results highlight the relevance of the physical environment to outdoor activities (Haug et al., 2008), but also reinforce the urge to understand the phenomenon using a more holistic approach (Bauman et al., 2012).

Outdoor activities are related to positive benefits to physical and mental health (Coventry et al., 2021). Among the activities performed in outdoor spaces, running has been pointed out as one of the most practiced and also has been contextualized as an important strategy to improve physical activity at a population level (WHO, 2019). Running is considered a sustainable activity, in which the costs associated with the training are relatively low, as well as non-expensive equipment is required. Despite these positive factors, more information is necessary about the interplay runner-environment in Brazilian macro-regions.

Since the relationship between the subject and environment is non-linear by nature, we used a network model to offer insights regarding the relative importance of individual and environmental variables to the running commitment (training volume and running pace) among different Brazilian contexts. It is a second step of a previous research study, in which we found that green spaces are positively related to a higher training volume among female runners (Thuany et al., 2022). Despite these results, the previous publication was focused on women runners and did not address the complex association between environmental factors. Considering the within-country differences, it is also important to understand the specificities of each region, what was not previously addressed. We hypothesized that the network topology will present different configurations between Brazilian macro-regions; and that regions with the best environmental indicators (i.e., higher green spaces, higher public illumination, percentage of the sidewalk, and pavement) will present a stronger influence on running commitment.

## 2. Materials and methods

### 2.1. Design and sample

The study presents a cross-sectional design in which data from the InTrack Project is used (InTrack, 2021). The project was developed with amateur Brazilian runners of both sexes, and data were collected between November 2019 and March 2020. Eligibility criteria included: self-classification as a runner, confirmation of participation in the study, and answering all mandatory questions in the questionnaire. The present study did not consider runners below 18 years and participants who did not answer all mandatory questions. All the participants were informed about the study’s purpose, risks, and ethical aspects. The study was performed following the Declaration of Helsinki and was approved by the Ethics Committee of the Federal University of Sergipe, Brazil (protocol no 3558630).

### 2.2. Data collection

The questionnaire “Profile characterization and associated factors for runner’s performance” (Thuany et al., 2020) was used. The questionnaire was transcribed to the Google Forms platform and sent as a link to the runners. This strategy covered all Brazilian states and did not aim to obtain a representative sample. Additionally, the questionnaire was disseminated through social media (Instagram, Facebook, and WhatsApp), and participants were encouraged to invite other runners to participate. The questionnaire’s validity information can be checked in Thuany et al. (2020). For the present study, the following information was used:

#### 2.2.1. Individual characteristics

Age (years), sex (male; female), socio-economic level–SES–(1 minimum wage; >1 to 3 wages; >3 to 5 wages), perception about the influence of weather characteristics and built environment in training commitment (yes; no), place of residence (capital of the state; not residence in the capital of the state), running training volume (km/week), and running pace (s/km). Information about socioeconomic level was based on minimum values of 2019–R$998,00; 193,22US) (Brasil, 2019).

#### 2.2.2. Environmental indicators

Information regarding environmental characteristics were obtained from the Brazilian Institute of Geography and Statistics, based on the Census 2010–Urban households’ characteristics and surroundings (IBGE, 2012). The total percentage of these characteristics was computed based on the total number of domiciles where these characteristics were reported and the total population [(number of residents which provided all parameters/total residents) × 100]. For the present study, we used information about public illumination (presence of at least one public light point, such as a streetlight, or lamps near the residence); pavement (presence of paved surfaces in public venues/streets); sidewalk (presence of paved sidewalks for pedestrians) and percentage of green areas (presence of trees along the sidewalks or tree beds that split lanes).

### 2.3. Statistical analysis

Descriptive statistic was presented as mean (SD) and frequency (%). Network analysis was performed to evaluate the association between individual and environmental characteristics. The EBICglasso parameter (Extended Bayesian Information Criterium) was used for network estimation. Centrality indicators (closeness, betweenness, and expected influence) were reported. Closeness values show the average distance between nodes, whereas those with higher closeness scores are more dependent on the network. The betweenness indicates the frequency to which a node lies on the shortest path connecting everyone else in a network. High values indicate that these nodes are important connection points between others in the network. For the expected values, variables with the highest values are more sensitive to change and can act as a hub by connecting other pairs of variables on the network (Hevey, 2018). Entropy values were calculated to verify the organization of the systems, for each macro-region. Color intensity is proportional to the strength of the association, while dashed lines show negative correlations. Statistical analyses were performed in JASP (Jeffreys’s Amazing Statistics Program), considering *p* < 0.05.

## 3. Results

Descriptive information is presented in Table 1 for the five Brazilian macro-regions. Most of the athletes are male and aged between 30 to 40 years. The highest frequency of participants reports an economic status between “1 and 3 minimum wages,” except for runners from the Mid-West, in which most of the participants reported “>3 and 5 minimum wages.” Most of the participants reported that weather and the built environment were factors that influenced training and practice commitment. For the environmental characteristics, sidewalks and green spaces are indicators with higher differences between regions. For both variables, the Southeast region presented the highest values.

**TABLE 1:** Participants’ descriptive information based on the macro-region of residence [mean ± SD; frequency (%)].

	North (*n* = 7.3%)	Northeast (*n* = 35.7%)	South (*n* = 12.4%)	Southeast (*n* = 36.3%)	Mid-west (*n* = 8.3%)
**Individual characteristics**					
**Sex**					
Female	39 (46.4%)	147 (35.8%)	62 (43.4%)	152 (36.4%)	40 (42.1%)
Male	45 (53.6%)	264 (64.2%)	81 (56.6%)	266 (63.3%)	55 (57.9%)
Age, years	35.6 (10.5)	37.40 (9.7)	36.87 (8.6)	38.99 (8.90)	39.50 (9.7)
**SES**					
Until 1 minimum wage	8 (9.5%)	33 (8%)	4 (2.8%)	19 (4.5%)	1 (1.1%)
>1 and 3 wage	45 (53.6%)	181 (44%)	64 (44.8%)	221 (52.9%)	31 (32.6%)
>3 and 5 wage	30 (35.7%)	189 (46%)	71 (49.7%)	174 (41.6%)	62 (65.3%)
>5 wage	0 (0%)	4 (1.0%)	0 (0%)	0 (0%)	0 (0%)
Missing	1 (1.2%)	4 (1.0%)	4 (2.8%)	4 (1.0%)	1 (1.1%)
**Perception about weather**					
No	27 (32.1%)	126 (30.7%)	30 (21.0%)	158 (37.8%)	30 (31.6%)
Yes	57 (67.9%)	284 (69.1%)	113 (79.0%)	260 (62.2%)	65 (68.4%)
Missing	0 (0%)	1 (0.2%)	0 (0%)	0 (0%)	0 (0%)
**Perception about built environment**					
No	26 (31.0%)	124 (30.2%)	27 (18.9%)	106 (25.4%)	16 (16.8%)
Yes	58 (69.0%)	287 (69.8%)	116 (81.1%)	312 (74.6%)	79 (83.2%)
Training volume	33.9 (21.9)	30.31 (23.1)	34.06 (24.5)	40.76 (36.16)	38.28 (30.7)
Running pace	325.1 (69.8)	329.10 (56.3)	310.55 (52.0)	322.62 (56.41)	329.01 (63.2)
**Environmental characteristics**					
Illumination	93.4 (3.8)	96.75 (0.7)	94.97 (1.6)	96.77 (1.9)	97.02 (2.1)
Pavement	74.9 (12.7)	84.37 (6.7)	89.90 (3.8)	95.88 (2.4)	84.61 (12.8)
Sidewalk	38.5 (14.2)	79.43 (9.2)	65.47 (4.6)	89.76 (3.9)	74.03 (9.5)
Green spaces	57.0 (21.5)	55.89 (9.8)	68.36 (17.7)	73.32 (5.7)	53.46 (25.6)

SES–Socioeconomic level: Based on value for 2019 (998,00R$; 193,22US); training volume (km/week); running pace (s/km).

**FIGURE 1:**
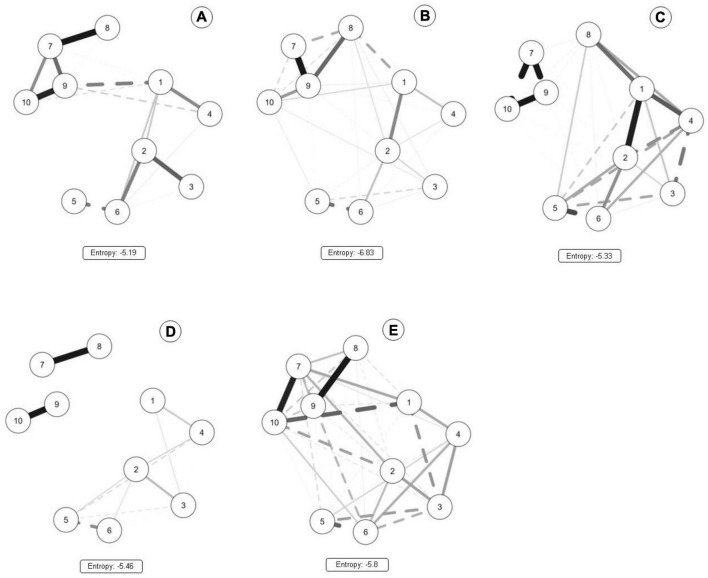
Network analysis results for Brazilian runners by region [**(A)** North; **(B)** Northeast; **(C)** South; **(D)** Southeast; **(E)** Mid-west]. 1: Capital residence; 2: SES; 3: Perception about the weather; 4: Perception about the built environment; 5: Running volume/week; 6: Running pace; 7: Illumination; 8: Pavement; 9: Sidewalk; 10: Green spaces.

The network plot, for each Brazilian macro-region, is presented in Figure 1. In a visual inspection, different network topologies are seen. Variables are sparser in panels d (Southeast) and c (South). For the Northeastern region (panel b), a positive and strong association between environmental indicators (illumination and sidewalks; pavement and sidewalks), as well as a negative relationship between the illumination and pavement. Therefore, for Northeast and Mid-West networks (panels b, e), individual and environmental characteristics are better linked. At the same time, for Southeast (panel d), these variables were less connected. For the North (panel a) and South regions (panel c), sidewalk and pavement variables were hubs between individuals and their environment.

Centrality results showed that illumination presented the highest values for expected influence in runners from the North and Mid-West regions (1.74 and 1.56). Similar results were shown for the Northeast and Southeast, in which sidewalks present higher values for expected influence (2.13; 0.91). For the South, to reside in the capital of the state presented a higher expected influence (1.67). Betweenness centrality results indicated similar results in North, Northeast, and Mid-West, where to reside in the capital presented higher values, indicating that this variable acts as a hub between variables. For the South and Southeast, pavement and training volume presented higher values. Entropy data suggested that the Northeast network was the most organized system (−6.8), followed by the Mid-West (−5.8), Southeast (−5.4), South (−5.3), and North (−5.1).

## 4. Discussion

The purpose of this study was to investigate the interplay between runners and their environment using a network approach. Our main findings showed that for the North and Mid-West regions, public illumination presents the highest values for the expected influence. In contrast, sidewalks present the highest values for the Northeast and Southeast. For betweenness centrality, in North, Northeast, and Mid-west, to reside in the capital of a state presents a hub, while pavement and training volume were important variables for South and Southeast.

The differences in the network topologies between the macro-regions confirm our first hypothesis. These results suggest that in continental size countries, such as Brazil, India, and China, where geographical, political, and cultural divisions exist, a more appropriate approach includes considering these differences but also considering sexes differences. A previous study using data from the InTrack project showed that for Brazilian women, green spaces were an important variable for training commitment (Thuany et al., 2022). These results differ from the current findings. Despite using the same project, the current study presents some advances compared to the previous publication, especially considering the theoretical bases. In this study, we embrace the complex systems approach as our guiding theory, complemented by network analysis to visually represent the interplay between individual and environmental variables. Although prior findings hold valuable practical implications within the Brazilian context, it is now crucial to shift focus towards comprehending the intricate relationships among variables, and not only the direct association between some factors, as usually considered in the scientific context. In addition, the substantial variability observed among the five macro-regions accentuates the importance to address these differences.

Especially for the environmental characteristics, a previous study showed that illumination, pavement, and cycle lanes were associated with physical activity patterns in adolescents from Rio Grande do Sul (South region) (da Silva et al., 2017). A national report showed that the North and Northeast regions presented the highest values for physical activity in leisure time (Brasil, 2015). Both regions present the highest average annual temperatures within-country (Kaplan and Chalfin, 2021). These characteristics can be related to network centralities. For the North and Mid-West, public illumination presented the highest values for expectable influence, which can be related to the security perception of physical activity in outdoor spaces (Kaplan and Chalfin, 2021). For betweenness results, residing in the capital of the state was the main variable for the North, Northeast, and Mid-West regions, indicating a relationship with the physical environment. Although Brazilian capital cities present better infrastructure than other cities from the states (IBGE, 2012), and most of the participants of the study reported residing in the capital of the state, these results should be considered carefully and face some limitations. Environmental information is related to the state’s capital, which does not represent the reality of all Brazilian cities. Most of the participants self-reported that weather and built environment are factors that influence training and practice commitment. Despite the relevance of the physical structure, weather characteristics are not controlled by participants, and can also affect the activities in outdoor spaces.

The environmental aspects that showed greater relationship strength in the networks have a close connection with the investment and development of the urban centers physical structure. Concerning public illumination (North and Mid-West), the arrangement of brighter environments can bring positive impacts such as a greater sense of security and more space for the practice of early morning (5 h 3 a.m. to 7 a.m.) and late evening physical activity since these tend to be part of the day when most people are available to get engaged in leisure physical activity due to the working hours. Sidewalks, besides being a service of the infrastructure of pedestrian circulation in the routine displacement of the cities, can be a safe space for sports, as in the case of running. Allocating spaces for sidewalks, as well as preserving their extension (i.e., corrective hole maintenance and unevenness, obstruction inspection), can provide a more appropriate and attractive built environment for outdoor physical activities. As previously stated, Brazilian territorial dimensions and regions specificity suggest a contextual evaluation to promote changes in the physical environment and availability of sports practice (Brasil, 2016). Large-scale changes in the built environment–such as improving public illumination and sidewalks’ quality–depend on public resources, public policies, and policymakers. However, since physical activity practice has an important relationship with health outcomes (Kapoor et al., 2022; Stevinson et al., 2022), this becomes a fundamental investment at individual and population levels.

This study is not free of limitations. Firstly, there is an important temporal gap between the subjects and the environmental information used. However, data about environmental characteristics were the most updated available at the moment. Sample size differences between the five macro-regions are related to data collection procedures. This is an important limitation, given that Brazil is a large country, with an estimated 2 million runners. Since our data collection procedures were based on a web survey, the results are based on data from runners that friendly decided to take part in this project, which can bias the results and impair the generalization of these findings. Third, information regarding the place of training is lacking. Our suggestion for future studies is to include the use of qualitative approaches to better understand how the physical environment of the cities influences activities in outdoor spaces, as well as what runners consider the most important changes that must be done in the physical environment.

## 5. Conclusion

As shown, public illumination (North and Mid-West) and sidewalks (Northeast and Southeast) are the most important variables to the runners’ network. In the South and Southeast regions, the running volume was important for the networks’ topology, while in the other regions to reside in the capital of the state was important to link variables within the network.

## Data availability statement

The raw data supporting the conclusions of this article will be made available by the authors, without undue reservation.

## Ethics statement

All the participants were informed about the study’s purpose, risks, and ethical aspects. The study was performed following the Declaration of Helsinki and was approved by the Ethics Committee of the Federal University of Sergipe, Brazil (protocol no. 3558630). The patients/participants provided their written informed consent to participate in this study.

## Author contributions

MT: conceptualization. MT and TG: methodology. MT and PB: formal analysis. MT and DV: writing—original draft preparation. KW, BK, and TG: writing—review and editing. All authors have read and agreed to the published version of the manuscript.
